# Validation of the Dutch Sexual Consent Scale-Revised

**DOI:** 10.1007/s10508-026-03415-3

**Published:** 2026-04-10

**Authors:** M. A. Van Stam, T. P. Humphreys, H. de Graaf, R. H. J. Scholte

**Affiliations:** 1 Radboud University, Behavioural Science Institute, Thomas van Aquinostraat 4, 6525 GD Nijmegen, The Netherlands; 2 Department of Psychology, Trent University, Peterborough, ON Canada; 3 Rutgers, the Dutch Expert Centre on Sexuality, Utrecht, Netherlands

**Keywords:** Sexual consent, Affirmative consent, Young adults, Sexual Consent Scale-Revised

## Abstract

Societal awareness of the importance of discussing and establishing sexual consent prior to sexual interaction is increasing. However, knowledge about Dutch young people’s attitudes and behaviors related to sexual consent remains limited and a validated Dutch sexual consent scale is currently lacking. To address these gaps, the dimensionality and construct validity of the Dutch Sexual Consent Scale-Revised (SCS-R) were evaluated in a cross-sectional quantitative study among Dutch adolescents and young adults aged 16–25 (*n* = 555), including heterosexual (*n* = 373) and non-heterosexual (*n* = 182) individuals, as well as cisgender women (*n* = 378), cisgender men (*n* = 150), and non-cisgender respondents (*n* = 27). Three of the five Dutch SCS-R subscales—Positive attitude; Perceived Behavioral Control; and Awareness—demonstrated psychometric robustness and can be applied in their original form within a sexuality- and gender- diverse Dutch context. For the remaining two subscales—Consent Norms and Indirect Consent Behaviors—correlated two-level models are suggested to improve applicability in the Dutch context. Another key finding of this study is that sexual consent experiences were significantly less aligned with sexual consent attitudes among cisgender women and non-heterosexual individuals, compared to cisgender men and heterosexual individuals. In conclusion, the results support the use of the Dutch SCS-R in future research, although adaptions are suggested for two of the subscales. In addition, the results underscore the importance of developing inclusive, context-sensitive policies, educational materials, and campaigns that empower young people to respect others’ boundaries and express their own preferences regarding sexual consent and activity.

**Supplementary Information:**

The online version contains supplementary material available at 10.1007/s10508-026-03415-3.

## Introduction

Public awareness of the necessity of discussing and obtaining sexual consent before engaging in sexual activity is increasing. This trend is exemplified by the transition in rape law from a coercion-centric framework (requiring the presence of “coercive circumstances” for the act to qualify) to a consent-based model (focused on the presence or absence of “unequivocal and voluntary agreement”; Dowds, 2020). The introduction of consent-focused laws aims to simplify the process of filing reports and securing convictions, ultimately aiming to reduce the substantial number of victims of rape, sexual assault, and other forms of sexual violence. These victims often endure profound and lasting physical and psychological consequences (Mason & Lodrick, 2013; Sugar et al., 2004).

This shift from coercion-centric to consent-focused rape laws has been implemented in numerous countries, including Ireland (1981), Belgium (1989), the United Kingdom (2003–2009), Turkey (2014), and Sweden (2018; Dowds, 2020). In the Netherlands, this legislative change was officially mandated in 2023 and came into force in July 2024 (Dutch Parliament, 2023). As a result, discussing and obtaining sexual consent before engaging in sexual activity should become the new norm. However, knowledge of young Dutch people’s attitudes towards asking for and giving sexual consent, as well as their actual behaviors, remains notably limited (Anyadike-Danes et al., 2024).

This knowledge is crucial for determining the need for additional interventions, such as public campaigns or educational programs, to normalize discussing sexual consent (Fenner, 2017; Muehlenhard et al., 2016). Furthermore, understanding attitudes and behaviors related to sexual consent, as well as the factors influencing them, can help identify specific target groups or situations where discussing sexual consent is not yet normalized and tailor interventions to their unique needs and priorities (Epps et al., 2023; Rabbitte, 2020). For example, campaigns could target college students or social settings like parties (Muehlenhard et al., 2016). Currently, however, no validated Dutch measures are available to provide insight into the sexual consent norms and behaviors of Dutch young people.

### Sexual Consent Scale-Revised

Internationally, one of the most frequently employed instruments in assessing attitudes, beliefs, and behaviors pertaining to the negotiation of sexual consent between partners is the Sexual Consent Scale-Revised (SCS-R; Humphreys & Brousseau, 2010). The SCS-R comprises five distinct subscales: (1) Positive Attitude Toward Establishing Consent (reflecting favorable beliefs about establishing consent before sexual activity commences); (2) (Lack of) Perceived Behavioral Control (indicating the extent of behavioral control individuals perceive in their negotiations of sexual consent); (3) Sexual Consent Norms (capturing beliefs about the preferred standards surrounding discussions of consent, and their stability over time and in relationships); (4) Indirect Consent Behaviors (assessing the current use of non-verbal methods in discussing consent); and (5) Awareness of Consent (measuring general awareness and discussions of consent among peers or partners).

Humphreys and Brousseau (2010) applied the theory of planned behavior (TPB; Ajzen, 1991) as a guiding framework for developing the SCS-R. In the TPB, intentions are described as predictors of behavior—in this context, negotiating consent with a sexual partner. The stronger the intention, the more likely a person is to directly or indirectly negotiate consent. Subsequently, intentions are determined by three independent factors in the TPB: attitude toward the behavior (Positive Attitude Toward Establishing Consent) subjective norms (Sexual Consent Norms and Awareness of Consent), and perceived behavioral control (Lack of Perceived Behavioral Control and Indirect Consent Behaviors). These three components were used to add and remove items from the initial SCS, developed by Humphreys and Herold (2007) to ensure adequate coverage of the TPB (Humphreys & Brousseau, 2010).

Notably, the SCS-R has been successfully translated and validated in Spanish and Japanese (Alfonso et al., 2021; Mukai et al., 2024), with several other translation and validation studies currently in progress (T. Humphreys, personal communication, April 9, 2024). Extending this process to include a Dutch translation and validation of the SCS-R holds significant potential for enhancing our understanding of young Dutch people’s attitudes, beliefs, and behaviors related to sexual consent. For example, future studies could use this instrument to identify specific target groups that would benefit from interventions aimed at normalizing discussions of sexual consent before engaging in sexual activity. Additionally, translating and validating an established, widely used scale will enable cross-national and cross-cultural comparisons. Such comparisons could, for example, help examine the beliefs, attitudes, and behaviors of Dutch young people relative to their peers in countries or schools where affirmative consent policies have been successfully introduced (Muehlenhard et al., 2016).

Crucial to studies examining sexual consent—and, therefore, to validating a sexual consent measure—is the inclusion of a diverse sample that encompasses individuals with varied gender identities (e.g., cisgender and non-cisgender individuals) and sexual orientations (e.g., heterosexual, homosexual, and bisexual individuals). This is essential because gender identity and sexual orientation are strongly associated with sexual consent-related attitudes and experiences (Mastrantonio et al., 2025; Muehlenhard et al., 2016). For instance, McKenna et al. (2021) found that nonbinary individuals reported more adaptive consent attitudes than cisgender individuals, while Martin-Storey et al. (2018) reported that gay, lesbian, and bisexual individuals were more likely to have experienced nonconsensual sexual encounters than heterosexual individuals. Consequently, to ensure a measure is applicable to gender- and sexually diverse populations, it is crucial to validate it using a diverse sample.

### Present Study

In summary, while the societal acknowledgment of the importance of discussing and attaining sexual consent is widespread, a validated and reliable measure to assess attitudes, beliefs, and behaviors regarding sexual consent is currently lacking in the Netherlands. Additionally, previous research underscores the importance of incorporating a diverse sample—including individuals from gender and sexual minority groups—when validating sexual consent measures (Anyadike-Danes et al., 2024; McKie et al., 2020). Therefore, the present study aims to address three key objectives. The primary goal is to examine the psychometric properties of the Dutch SCS-R within a sample of young adults (ages 16–25) in the Netherlands. Second, the study aims to initially explore the construct validity of the Dutch SCS-R by testing the TPB-based hypothesis that the SCS-R subscales are associated with sexual consent behaviors. Third, both internal consistency and construct validity will be explored separately in subgroups based on gender identity and sexual orientation.

## Method

### Participants

Respondents who did not answer any of the SCS-R items were excluded from the analytic sample. Consequently, the final analytic sample comprised 555 respondents. Detailed demographic information, categorized by sexual orientation and gender identity, is presented in Table 1 and Table A in the Electronic Supplement.

**Table 1:** Demographic information by gender identity and sexual orientation and for the total sample

Variable	Heterosexual (*n* = 373)		Non-heterosexual (*n* = 182)		Full sample (*n* = 555)	
	*n*	%	*n*	%	*n*	%
Age group (in years)						
16–17	22	6	11	6	33	6
18–19	91	16	22	12	83	15
20–21	104	28	54	30	158	28
22–23	115	31	67	37	182	33
24–25	71	19	2	15	99	18
Sex assigned at birth						
Female	253	78	143	79	396	71
Male	120	32	39	21	159	29
Gender identity^a^						
Cisgender women	250	67	128	70	378	68
Cisgender men	117	31	33	18	150	27
Non-cisgender	6	2	21	12	27	5
Sexual orientation						
Heterosexual	373	100	0	0	373	67
Heteroflexible	0	0	27	15	27	5
Bisexual	0	0	60	33	60	11
Lesbian/Gay	0	0	28	15	28	5
Questioning	0	0	38	21	38	7
Other^b^	0	0	29	16	29	5
Relation status						
Not in a relation	73	20	93	51	280	51
In a relation	300	80	89	49	275	49
Sexual experience^c^						
No	73	20	37	20	110	20
Yes	300	80	145	80	445	80
Educational level^d^						
Level 1–4	91	24	47	26	138	25
Level 5–8	282	76	135	74	417	75
Housing type						
With parents	177	48	61	34	238	43
Student room/studio	146	39	94	52	240	43
Own (rental) house	46	12	24	13	70	13
Daily occupation						
Education	283	76	142	78	425	77
Work	87	23	37	20	124	22
No work or education	3	1	3	2	6	1
Cultural background^e^						
Dutch/Western	347	93	176	97	523	95
Non-Western (+ Dutch)	21	6	4	2	25	5
Importance of religion						
Not (at all)	194	52	98	54	292	53
A little	70	19	55	30	125	22
Very	109	29	29	16	138	25

The number of respondents and percentages do not sum up to 555/100% when the variable contained missing data

^a^Cisgender women were defined as respondents who were assigned female at birth and currently identify as women. Cisgender men were defined as respondents who were assigned male at birth and currently identify as men. Non‑cisgender respondents were defined as respondents whose current gender identity did not align with their sex assigned at birth. These respondents identified as transgender women (*n* = 2), transgender men (*n* = 7), nonbinary (*n* = 4), bigender (*n* = 7), questioning (*n* = 6), or other (*n* = 1)

^b^The category “other” in Sexual Orientation included respondents that identified as pansexual (*n* = 12), Queer (*n* = 12), Asexual/demisexual (*n* = 5)

^c^Sexual experience was operationalized as “actions that are intended to sexually stimulate someone, such as kissing, touching genitals, oral sex, or vaginal or anal sex”

^d^International Standard Classification of Education (ISCED) educational level 1–4 = primary; lower secondary; upper secondary; post-secondary non-tertiary; and ISED educational level 5–8 = short cycle tertiary; bachelor or equivalent; master or equivalent; doctoral or equivalent

^e^Cultural background categories are operationalized as: Dutch/Western = Only Dutch (*n* = 511), Dutch and North America (*n* = 1), Dutch and Western Europe (11); and “Non-Western (+ Dutch)” as Dutch and Turkish (*n* = 4), Dutch and Moroccan (*n* = 1), Dutch and Surinamese (*n* = 4), Dutch and Antillean (*n* = 2), Dutch and Europe non-Western (*n* = 3), Dutch and South/West Asia (*n* = 7), Dutch and North/East/Central Asia (*n* = 6), Dutch and not specified (*n* = 4)

The age of the respondents ranged from 16 to 25 years (*M* = 21.33; *SD* = 2.24). The majority identified as cisgender women (*n* = 378, 68%), 27% of the respondents as cisgender men (*n* = 150), and 5% (*n* = 27) as non-cisgender (i.e. transgender female, transgender male, nonbinary; bi-gender; questioning; or “other”). More than half of the respondents (67%, *n* = 373) described their sexual orientation as heterosexual, followed by bisexual (11%, *n* = 60), questioning (7%, *n* = 38), and the remaining respondents as heteroflexible (5%, *n* = 27), lesbian or gay (5%, *n* = 28), or other (5%, *n* = 29). About half (49%, *n* = 275) of the respondents had a partner, and the majority (80%, *n* = 445) had previous sexual experiences.

Regarding primary daily occupation, 77% (n = 425) of respondents were attending education, while 22% (*n* = 124) reported working or combining work with education. Additionally, six respondents (1%) reported neither working nor attending school. Among the 425 respondents still engaged in education, 47 (11%) were attending high school, 34 (6%) were enrolled in secondary vocational education, 152 (36%) were pursuing higher vocational education, 190 (45%) were university students, and two respondents indicated participation in another form of education. For international comparisons, a composite variable was created by combining the “highest completed level of education” and the “current level of education” for respondents primarily engaged in education. This composite variable ranged from primary school (ISEC level 1) to university (ISEC level 7). Details of this distribution are presented in Table 1.

A substantial majority of the respondents (95%, *n* = 523) reported a Dutch or Western cultural background. Additionally, 25% (*n* = 138) indicated that religion was very important in their lives.

### Procedure

The data for this study were gathered from a cohort of 686 Dutch young adults. Inclusion criteria required participants to be between 16 and 25 years old (these ages serve as arbitrary markers of the developmental and social transitions that characterize young adulthood) and sufficiently fluent in Dutch to comprehend the questions. This research was part of a broader study on diversity and sexual health among young adults in the Netherlands, which included questions about participants’ socio-demographic characteristics, personality, self-esteem, and experiences and attitudes related to sexual consent. The cross-sectional quantitative study was conducted in May 2023.

Respondent recruitment was conducted through diverse online platforms, encompassing social media pages, newsletters of youth associations or groups related to sports, culture, religion, and LGBTQ+, as well as the researchers’ social media channels. Additionally, the University Participation’s online research system (SONA) was utilized for recruitment. An a priori minimum of 400 participants who completed the questionnaire was established to ensure sufficient power for our analyses. However, recruitment was more successful than anticipated, resulting in 686 participants.

Prior to beginning the questionnaire, all respondents were informed about the research and willingly provided digital informed consent. To safeguard respondents’ privacy, contact information was not requested, and broad answer categories were presented, ensuring anonymity during the subsequent data analysis phase. Individuals who utilized the online research system had the option to receive a reward of 0.5 study points, whereas other respondents did not receive any compensation for their involvement.

### Measures

#### Sexual Consent Scale-Revised

The SCS-Revised is a 39-item, Likert-type measure, developed by Humphreys and Brousseau (2010), that measures an individual’s attitudes and behaviors concerning how sexual consent should be, and is, discussed between sexual partners. The SCS-R consists of five subscales: 1. Positive Attitude Toward Establishing Consent (e.g., “I feel that sexual consent should always be obtained before the start of any sexual activity”, α = 0.84 in the English SCS-R); 2. (Lack of) Perceived Behavioral Control (e.g., “I would have difficulty asking for consent because it would spoil the mood”, α = 0.86); 3. Sexual Consent Norms (e.g., “I think that obtaining sexual consent is more necessary in a new relationship than in a committed relationship”, α = 0.67); 4. Indirect Consent Behaviors (e.g., “Typically I communicate sexual consent to my partner using nonverbal signals and body language”, α = 0.78); and 5. Awareness of Consent (e.g., “I have not given much thought to the topic of sexual consent”—reverse-coded, α = 0.71).

In the English SCS-R, all items are rated on a seven-point Likert scale ranging from 1 (strongly disagree) to 7 (strongly agree). In the Dutch SCS-R, two additional response options were included: “I prefer not to answer this question.” (8)—added in accordance with Ethical Committee regulations—and “Not applicable” (9)—for items referencing a “current (or most recent) partner” or “previous sexual experiences,” as not all respondents had a partner (before) or sexual experience. The Dutch translation of the SCS-R items is available in the Electronic Supplement (Table B), and the full version can be requested from the first author. Responses marked as 8 or 9 were recoded as missing values in the analyses.

The Dutch translation of the SCS-R followed the translation guidelines of Beaton et al. (2000). First, two native Dutch speakers independently translated the measure, after which an English language expert performed a back-translation. Both the Dutch translation and back-translation were reviewed—initially by the first author and subsequently by two experts specializing in sexual consent and sexual development. Discrepancies were resolved through discussions, mainly by modifying formal or overly lengthy phrases to better reflect commonly used Dutch language. For example, the original item “I have heard sexual consent issues being discussed by other students on campus” was translated as “I have heard peers talk about topics related to sexual consent.”

#### Sexual Consent Experiences

Questions related to sexual consent experiences were formulated based on the Sexual Experiences Survey (Burke et al., 2020), to assess respondents’ previous experiences with sexual consent during past encounters. In the construct analysis, the aim was to test the TPB-based hypothesis that the SCS-R subscales were associated with the behavior of negotiating sexual consent before a sexual encounter. Therefore, the following item was incorporated in the construct validity analysis: “I have had sexual experiences where I wanted to have sex and had given or asked for permission (using words or body language).” The item was measured using a five-point Likert scale with the following answer options: No, never (0); One or two times (1); Three times or more (2); Prefer not to answer (3); and Not applicable (4). Responses (3) and (4) were recoded as missing values for analysis. In the construct validity analyses, response options (1) and (2) were combined into the category “yes, once or more.”

#### Demographic Variables

Questions related to respondents’ backgrounds encompassed demographic, sexual and relational, educational, occupational, housing, cultural, and religious aspects. Details about the demographic variables which were based on single items, are presented in Table 1 and Table A in the Electronic Supplement. Questions and answer options were based on previous research by de Graaf et al. (2017). The following variables (Gender Identity and Sexual Orientation) were composed by combining several questions or answer categories.

Gender identity was operationalized by combining the item assessing sex assigned at birth (response options: Female; Male; Intersex) with the item assessing current gender identity (response options: Woman; Man; Woman and man; Woman and man, but more woman; Woman and man, but more man; Not a man or a woman; I don’t know yet; I prefer not to answer; My answer is not listed). Respondents who were assigned female at birth and currently identified as women were classified as cisgender women. Respondents who were assigned male at birth and currently identified as men were classified as cisgender men. Respondents whose current gender identity did not align with their sex assigned at birth, or who selected I don’t know yet or My answer is not listed, were classified as non‑cisgender (Fraser, 2018). Footnote a of Table 1 provides the distribution of respondents across the individual gender identity categories.

Sexual orientation was operationalized based on the classification system of Legate and Rogge (2019). Entailing a combination of the following two items: respondents’ self-reported sexual orientation (answer options*:* Hetero; Lesbian/homo/gay; Bisexual; Pansexual; Queer; Asexual/demisexual; I don’t know yet; I don’t want to answer and; My answer is not listed) and the item assessing respondents’ degree of sexual attraction (answer options: Only men; Both men and women, but mostly men; Both men and women equally; Both men and women, but mostly women; Only women; Not to women or men; I don’t know yet and; My answer is not listed). In the analyses, “Other” in Sexual Orientation included respondents who identified as “Pansexual”, “Queer”, and “Asexual or demisexual”, or selected “My answer is not listed”, to avoid categories with only a few respondents. The footnote of Table 1 contains information about the number of respondents within the separate categories. In the cases that the two answers did not correspond a category was manually assigned based on the respondent’s explanation (*n* = 5) or, when no explanation was provided, the most plausible option was selected following discussion among researchers. For example, “heteroflexible” for those respondents who did not identify as fully bisexual but answered being attracted to both men and women (e.g., being attracted to “Both men and women, but mostly men”; *n* = 27); or “Questioning” for respondents who answered “I don’t know yet” on one or both of the two questions (*n* = 4). This resulted in the following six categories within Sexual Orientation: Heterosexual; Heteroflexible; Bisexual; Lesbian/Gay; Questioning; and Other.

### Statistical Analysis

To address the research questions, three distinct analyses were conducted: 1. Confirmatory factor analysis (CFA) was performed to determine the factor structure of the Dutch SCS-R; 2. Internal consistency analysis to assess the reliability of the SCS-R scale; and 3. Independent samples *t*-test to explore the construct validity of the SCS-R scales. The second and third analyses were performed both in the overall sample and separately within the gender identity and sexual orientation subgroups. Further details are presented below. All statistical analyses were performed using IBM SPSS and AMOS version 29. A *p*-value of < 0.05 was considered statistically significant where applicable.

#### Confirmatory Factor Analysis

CFA was conducted to evaluate whether the 39 translated items adequately measure five latent factors in the Dutch setting: (1) Positive Attitude Toward Establishing Consent, (2) (Lack of) Perceived Behavioral Control, (3) Sexual Consent Norms, (4) Indirect Consent Behaviors, and (5) Awareness of Consent. To assess model fit, the following indices were used: Comparative Fit Index (CFI), Root Mean Square Error of Approximation (RMSEA), and Standardized Root Mean Square Residual (SRMR). The chi-square statistic was also reported but not used to determine model adequacy, as it tends to reject the null hypothesis of perfect fit in large samples. CFI values above 0.90 indicate adequate fit, and values above 0.95 indicate good fit. For RMSEA and SRMR, values below 0.08 suggest adequate fit, while values below 0.05 indicate good fit (Kline, 2016). In this study, RMSEA values are expected to be relatively high due to the known limitations of RMSEA in models with low degrees of freedom—such as the single-factor models analyzed here (Kline, 2016).

An overall conclusion of “acceptable model fit” is drawn when at least one absolute fit index (e.g., RMSEA or SRMR) and one incremental fit index (e.g., CFI) meet the threshold for adequate or good fit (Hu & Bentler, 1999). In cases where two or more fit indices fall below acceptable levels, the possibility of developing an adapted model is considered. Such adaptations are guided by both theoretical rationale and empirical criteria—for example, constructing a two-factor model in which items that are conceptually and statistically closely related (factor loading > 0.30) are grouped within a single factor.

The computation of mean subscale scores adhered to the methodology outlined in the SCS-R manuscript by Humphreys and Brousseau (2010). This process involved summing the scores of all valid items and dividing by the number of valid items for each subscale. Missing values were excluded from the calculation, and a minimum requirement of at least 50% valid items in the scale was necessary to compute a mean subscale score. The resulting mean item and subscale scores are detailed in Table 3.

#### Internal Consistency

Internal consistency was assessed using Cronbach’s alpha and McDonald’s omega for the SCS-R subscales. The values of these reliability coefficients lie between 0 and 1, with values above 0.70 generally considered acceptable (Gliem & Gliem, 2003; McDonald, 1999; Viladrich et al., 2017).

Internal consistency was assessed for the complete sample and separately for gender and sexual minority subgroups (Anyadike-Danes et al., 2024; Willis et al., 2019). Since a minimum sample size of 30 is recommended for estimating internal consistency (Bujang et al., 2018), reliability scores for non-cisgender respondents could not be reliably calculated due to statistical power concerns.

#### Exploration of Construct Validity

Given the absence of other validated sexual consent questionnaires in Dutch, construct validity was explored by testing the hypothesized relationship between the SCS-R subscales and respondents’ previous experiences with sexual consent. This hypothesis was based on the TPB, which guided the development of the SCS-R. Specifically, according to the TPB, respondents who “negotiated sexual consent before a sexual encounter at least once” were hypothesized to report: (H1) significantly greater Positive Attitude Toward Establishing; (H2) significantly lower Lack of Perceived Behavioral Control; and (H3) significantly greater Awareness of Consent.

The SCS-R subscales Sexual Consent Norms and Indirect Consent Behaviors were excluded from the construct validity analyses. These factors were not expected to correlate with the measure of “negotiating sexual consent, using words or body language, at least once,” as this measure does not distinguish between being in a relationship or not, nor between verbal and nonverbal consent. Consequently, individuals who are more (or less) flexible in their sexual consent norms over time and across relationships, or who rely more (or less) on body language when negotiating consent, would not necessarily report a higher (or lower) frequency of negotiating sexual consent using words or body language during their last sexual encounter.

These three hypotheses were examined using independent-samples *t*-tests for the total sample, as well as for gender and sexual minority subgroups separately. *T*-tests were preferred over binary logistic regression due to low events-per-variable ratios, which have been associated with poorer predictive performance upon validation (Van Smeden et al., 2019). However, due to a lack of statistical power (0.10–0.11) resulting from small subgroup sizes in the non-cisgender group (Serdar et al., 2021), the hypotheses could not be reliably tested in this group. Other assumptions underlying the independent-samples *t*-test—including normally distributed sampling distributions, homogeneity of variance, and independent scores—were examined and accepted unless reported otherwise (Kim & Park, 2019).

Effect sizes for the mean differences were calculated based on Cohen’s *d*. A Cohen’s *d* score of 0.2–0.5 was considered a small effect size; 0.5–0.8, a medium effect size; and greater than 0.8, a large effect size (Fritz et al., 2012).

## Results

### Confirmatory Factor Analysis

Model fit information for all tested subscales is presented in Table 3. Three of the five subscales met the predefined criterion for “overall acceptable fit”—defined as achieving threshold values for at least one absolute fit index and one incremental fit index. These subscales were: Positive Attitude Toward Establishing Consent, (Lack of) Perceived Behavioral Control, and Awareness of Consent.

For the subscales, Sexual Consent Norms and Indirect Consent Behaviors, all three fit indices indicated less than adequate model fit. Therefore, adapted versions of both subscales were examined—based on theoretical considerations and the factor loadings observed in the original scale—to improve model adequacy.

Acceptable overall model fit was achieved for Sexual Consent Norms when analyzed as a correlated two-factor model (see Tables 2 and 3). The first factor—Sexual Consent Norms: Relationship—included Items 23, 24, 25, and 28 addressing the stability of preferred standards for discussing consent over time and across relationship stages (e.g., “I think that obtaining sexual consent is more necessary in a new relationship than in a committed relationship”). These items also demonstrated factor loadings above 0.30 in the original model (see Table 3 for loadings in the two-factor model). The second factor—Sexual Consent Norms: Activity—comprised Items 26, 27, and 29, which focused on shifts in consent norms based on the type of sexual activity or the timing within the sexual encounter (e.g., “I believe it is enough to ask for consent at the beginning of a sexual encounter”). The two factors were weakly correlated (*r* = 0.39).

**Table 2:** Model fit parameters for each of the models tested

	*χ*^2^ (df)	CFI	SRMR	RMSEA
Good fit	*p* > 0.05	0.95–1.00	< 0.05	< 0.05
Adequate fit		0.90–0.95	< 0.08	< 0.08
Subscale				
1. Positive Attitude Toward Establishing Consent	166.989 (44)	**0.925**	**0.048**	**0.077**
2. (Lack of) Perceived Behavioral Control	157.753 (44)	**0.922**	**0.053**	0.092
3. Sexual Consent Norms	159.408 (14)	0.842	0.091	0.144
3a. Sexual Consent Norms—two factor model	99.833 (13)	**0.906**	**0.071**	0.116
4. Indirect Consent Behaviors	92.195 (9)	0.755	0.105	0.170
4a. Indirect Consent Behaviors—two factor model	31.288 (8)	**0.934**	**0.531**	0.095
5. Awareness of Consent	30.534 (2)	**0.929**	**0.071**	0.198

The chi-square statistic was statistically significant in all models at least at the 0.05 level. Fit indices in bold indicate adequate or good model fit

**Table 3:** Factor loadings, means, and standard deviations for the SCS-R items

Items		Loadings original	Loadings adapted^a^	*M*	*SD*
Subscale 1: Positive Attitude Toward Establishing Consent				5.27	0.90
1	I feel that sexual consent should always be obtained before the start of any sexual activity	0.72	–	5.42	1.46
2	I believe that asking for sexual consent is in my best interest because it reduces any misinterpretations that might arise	0.64	–	6.01	1.15
3	I think it is equally important to obtain sexual consent in all relationships regardless of whether or not they have had sex before	0.45	–	6.21	1.07
4	I feel that verbally asking for sexual consent should occur before proceeding with any sexual activity	0.57	–	4.56	1.51
5	When initiating sexual activity, I believe that one should always assume they do not have sexual consent	0.54	–	4.47	1.61
6	I believe that it is just as necessary to obtain consent for genital fondling as it is for sexual intercourse	0.60	–	5.95	1.24
7	Most people that I care about feel that asking for sexual consent is something I should do	0.61	–	5.19	1.43
8	I think that consent should be asked before any kind of sexual behavior, including kissing or petting	0.70	–	4.60	1.65
9	I feel it is the responsibility of both partners to make sure sexual consent is established before sexual activity begins	0.64	–	6.09	1.03
10	Before making sexual advances, I think that one should assume ‘‘no’’ until there is clear indication to proceed	0.68	–	5.20	1.45
11	Not asking for sexual consent some of the time is okay [R]	0.55	–	3.76	1.64
Subscale 2: (Lack of) Perceived Behavioral Control				2.88	1.15
12	I would have difficulty asking for consent because it would spoil the mood	0.83	–	3.60	1.81
13	I am worried that my partner might think I’m weird or strange if I asked for sexual consent before starting any sexual activity	0.81	–	3.14	1.80
14	I would have difficulty asking for consent because it doesn’t really fit with how I like to engage in sexual activity	0.81	–	3.29	1.80
15	I would worry that if other people knew I asked for sexual consent before starting sexual activity, that they would think I was weird or strange	0.62	–	2.47	1.53
16	I think that verbally asking for sexual consent is awkward	0.79	–	3.38	1.82
17	I have not asked for sexual consent (or given my consent) at times because I felt that it might backfire and I wouldn’t end up having sex	0.47	–	2.69	1.71
18	I believe that verbally asking for sexual consent reduces the pleasure of the encounter	0.80	–	3.07	1.69
19	I would have a hard time verbalizing my consent in a sexual encounter because I am too shy	0.56	–	2.95	1.62
20	I feel confident that I could ask for consent from a new sexual partner [R]	0.42	–	5.63	1.31
21	I would not want to ask a partner for consent because it would remind me that I’m sexually active	0.49	–	2.06	1.33
22	I feel confident that I could ask for consent from my current partner [R]	0.29	–	6.39	1.11
Subscale 3: Sexual Consent Norms				4.73^b^	0.91^b^
23	I think that obtaining sexual consent is more necessary in a new relationship than in a committed relationship	0.84	0.84^I^	5.31	1.70
24	I think that obtaining sexual consent is more necessary in a casual sexual encounter than in a committed relationship	0.91	0.91^I^	5.53	1.69
25	I believe that the need for asking for sexual consent decreases as the length of an intimate relationship increases	0.63	0.63^I^	4.82	1.57
26	I believe it is enough to ask for consent at the beginning of a sexual encounter	0.28	0.71^II^	3.99	1.52
27	I believe that sexual intercourse is the only sexual activity that requires explicit verbal consent	0.07	0.36^II^	2.24	1.27
28	I believe that partners are less likely to ask for sexual consent the longer they are in a relationship	0.36	0.36^I^	6.07	1.02
29	If consent for sexual intercourse is established, petting and fondling can be assumed	0.26	0.42^II^	5.12	1.49
Subscale 4: Indirect Consent Behaviors				5.00^c^	1.06^c^
30	Typically I communicate sexual consent to my partner using nonverbal signals and body language	0.32	0.64^I^	6.05	1.17
31	It is easy to accurately read my current (or most recent) partner’s nonverbal signals as indicating consent or non-consent to sexual activity	0.28	0.48^I^	5.04	1.52
32	Typically I ask for consent by making a sexual advance and waiting for a reaction, so I know whether or not to continue	0.13	0.30^I^	4.70	1.61
33	I don’t have to ask or give my partner sexual consent because my partner knows me well enough	0.81	0.81^II^	4.85	1.76
34	I don’t have to ask or give my partner sexual consent because I have a lot of trust in my partner to ‘‘do the right thing’’	0.82	0.84^II^	4.58	1.79
35	I always verbally ask for consent before I initiate a sexual encounter [R]	0.38	0.55^I^	3.08	1.61
Subscale 5: Awareness of Consent				5.24	1.43
36	I have discussed sexual consent issues with a friend	0.89	–	5.48	1.81
37	I have heard sexual consent issues being discussed by other students on campus	0.79	–	5.29	1.84
38	I have discussed sexual consent issues with my current (or most recent) partner at times other than during sexual encounters	0.40	–	5.26	1.92
39	I have not given much thought to the topic of sexual consent [R]	0.47	–	2.91	1.67

Items with [R] are reverse coded. ^I^Items loading on factor I within the two factor models; ^II^Items loading on factor II within the two factor models. ^a^For the subscales Sexual Consent Norms and Indirect Consent Behaviors adapted scale-two factor models-were developed because fit indices indicated less than adequate fit.; ^b^”Sexual Consent Norms: Relationship” *M* = 5.43, SD = 1.18; “Sexual Consent Norms: Activity” *M* = 3.78, SD = 1.00; ^c^Mean scores for the CFA based factors; “Indirect Consent Behaviors: Nonverbal” *M* = 5.14, SD = 1.06; “Indirect Consent Behaviors: Partner” *M* = 4.71, SD = 1.64

Acceptable overall model fit was also achieved for Indirect Consent Behaviors when analyzed as a correlated two-factor model (see Tables 2 and 3). The first factor—Indirect Consent Behaviors: Nonverbal—included Items 30, 31, 32, and 35, which addressed the typical use of nonverbal signals and body language to communicate sexual consent (e.g., “Typically I communicate sexual consent to my partner using nonverbal signals and body language”). The second factor—Indirect Consent Behaviors: Partner—comprised Items 33 and 34, focusing on indirect consent behaviors within the context of one’s current partner (e.g., “I don’t have to ask or give my partner sexual consent because my partner knows me well enough”). These items also demonstrated strong factor loadings (> 0.80) in the original model. The two factors were weakly to moderately correlated (*r* = 0.49).

### Internal Consistency

**Fig. 1:**
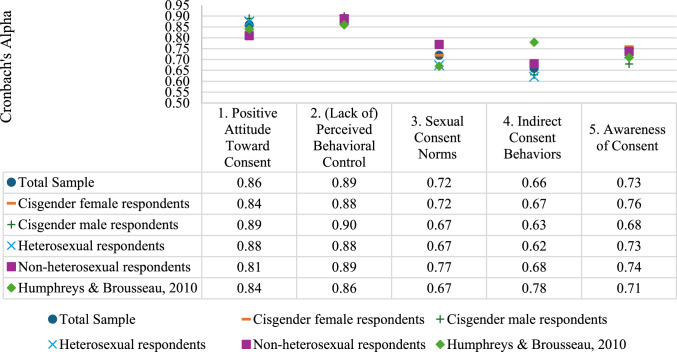
Cronbach’s alpha scores for the Dutch and English SCS-R (total sample and subgroups)

For the total sample, acceptable internal consistency was demonstrated across the SCS-R subscales (Fig. 1): Positive Attitude Toward Establishing Consent (α = 0.86; ω = 0.86); Lack of Perceived Behavioral Control (α = 0.89; ω = 0.90); Sexual Consent Norms (α = 0.72; ω = 0.72); Awareness of Consent (α = 0.73; ω = 0.72). Internal consistency was comparable to or higher than that reported for the English SCS-R (Humphreys & Brousseau, 2010), except for SCS-R Subscale 4 (Indirect Consent Behaviors; α = 0.66; ω = 0.63), which demonstrated a higher internal consistency in the English version (α = 0.78). Internal consistency scores among cisgender women, cisgender men, heterosexual, and non-heterosexual respondents were comparable, with a maximum difference of 0.05, and exhibited a similar pattern to that of the total sample (see Fig. 1).

Internal consistency of the additional subscales, as identified through the CFA analyses, was as follows: Sexual Consent Norms: Relationship (α = 0.79; ω = 0.83), Sexual Consent Norms: Activity (α = 0.48; ω = 0.53), Indirect Consent Behaviors: Nonverbal (α = 0.57; ω = 0.55), and Indirect Consent Behaviors: Partner (α = 0.82, ω could not be estimated because there are only two items in this scale). These results indicate that only Sexual Consent Norms: Relationship and Indirect Consent Behaviors: Partner demonstrated acceptable internal consistency.

### Construct Validity

To explore construct validity, the three a-priori formulated hypotheses about the relation between the SCS-R subscales and previous experiences with sexual consent were analyzed.

The results from the total sample support all three hypotheses (Table 4): respondents who “negotiated sexual consent before a sexual encounter at least once” reported: (H1) significantly greater Positive Attitude Toward Establishing Consent (*t*(433) = − 4.10, *p* < .001, *d* = − 0.82); (H2) significantly less Lack of Perceived Behavioral Control (*t*(423) = 3.96, *p* < .001, *d* = 0.80); (H3) and significantly more Awareness of Consent (*t*(363) = − 2.00, *p* = .02, *d* = − 0.45), compared to those who never negotiated sexual consent before a sexual encounter.

**Table 4:** Results of construct validity analyses examining the association between the SCS-R subscales and sexual consent experiences

SCS-R subscale	Never, discussed sexual consent		Once or more discussed sexual consent		*p*	Cohen’s *d*
	*M*	*SE*	*M*	*SE*		
Total sample						
Positive attitude toward consent	4.56	1.18	5.26	0.84	< .001*	0.86
Lack of behavioral control	3.66	0.95	2.76	1.13	< .001*	0.80
Awareness of consent	4.83	1.15	5.42	1.32	0.02*	− 0.45
Cisgender women						
Positive attitude toward consent	4.92	0.99	5.28	0.80	0.06	− 0.44
Lack of behavioral control	3.30	0.92	2.78	1.07	0.43	0.49
Awareness of consent	5.18	1.05	5.44	1.32	0.25	− 0.20
Cisgender men						
Positive attitude toward consent	4.16	1.28	5.15	0.99	< .001*	− 0.96
Lack of behavioral control	4.01	0.87	2.68	1.19	< .001*	1.15
Awareness of consent	4.36	1.16	5.27	1.37	0.03*	− 0.68
Heterosexual						
Positive attitude toward consent	4.32	1.12	5.19	0.87	< .001*	− 0.97
Lack of Behavioral Control	3.69	1.02	2.82	1.10	< .001*	0.79
Awareness of consent	4.69	1.09	5.32	1.32	0.03*	− 0.48
Non-heterosexual						
Positive attitude toward consent	5.89	0.27	5.40	0.77	0.11	0.63
Lack of behavioral control	3.47	0.51	2.64	1.17	0.08	0.72
Awareness of consent	5.64	1.38	5.61	1.30	0.49	0.02

^*^Hypothesis supported

Among cisgender women, none of the hypotheses were supported (Table 4): (H1) *t*(309) = − 1.61, *p* = .06, *d* = − 0.44; (H2) *t*(300) = − 1.72, *p* = .43, *d* = − 0.49; and (H3) *t*(265) =  − 0.68, *p* = .25, *d* = − 0.20. By contrast, for cisgender men respondents all hypotheses were supported: (H1) *t*(103) =  − 3.23, *p* < 0.01, *d* = − .96; (H2) *t*(102) = 3.89, *p* < .001, *d* = 1.15; and (H3) *t*(82) =  − 1.91, *p* = 0.03, *d* = − 0.68.

Similarly, all hypotheses were supported among heterosexual respondents (Table 4): (H1) *t*(290) =  − 4.47, *p* < .001, *d* = − 0.97; (H2) *t*(286) = 3.56, *p* < .001, *d* = 0.79; and (H3) *t*(240) =  − 1.94, *p* = .03, *d* = − 0.48. In contrast, none of the hypotheses were supported among non− heterosexual respondents; (H1) *t*(141) = 1.25, *p* = .11, *d* = 0.63; (H2) *t*(139) = 1.41, *p* = .08, *d* = 0.72; and (H3) *t*(121) = 0.04, *p* = .49, *d* = 0.02. However, these results have to be interpreted with caution due to the small number of non-heterosexual respondents who reported never discussing sexual consent (3%; *n* = 4).

## Discussion

The study aimed to explore the underlying dimensionality, reliability, and construct validity of the Dutch SCS-R among young adults aged 16–25 in the Netherlands. It also aimed to explore the scale’s reliability and construct validity across subgroups based on gender and sexual identity. Due to the lack of validated Dutch instruments for assessing beliefs, attitudes, and behaviors related to sexual consent, validating the SCS-R is a valuable first step in advancing our understanding of sexual consent dynamics in the Netherlands.

The results of the CFA and internal consistency analyses indicated that the Dutch versions of the subscales Positive Attitude Toward Establishing Consent, (Lack of) Perceived Behavioral Control, and Awareness of Consent can be reliably applied within a gender- and sexual identity-diverse Dutch context. For the subscales Sexual Consent Norms and Indirect Consent Behaviors, a two-factor model—rather than a single-factor structure—was suggested to improve their applicability in this setting. Furthermore, comparable internal consistency scores for all subscales were observed among cisgender women and cisgender men, as well as heterosexual and non-heterosexual respondents. Construct validity was supported through analysis of the associations between the SCS-R subscales and past sexual consent experiences within the total sample. However, statistically significant associations were found only among cisgender men and heterosexual individuals, and not among cisgender women and non-heterosexual individuals. This suggests that, among cisgender women and non-heterosexual individuals, sexual consent attitudes and intentions are less consistently aligned with sexual consent experiences. The implications of these findings are discussed below.

First, the findings support the use of the SCS-R subscales Positive Attitude Toward Establishing Consent, (Lack of) Perceived Behavioral Control, and Awareness of Consent within the Dutch context. These subscales demonstrated satisfactory psychometric properties and can be considered reliable tools for assessing sexual consent attitudes, behavioral intentions, and awareness among Dutch young adults across diverse gender and sexual identities. However, the results indicate that Sexual Consent Norms and Indirect Consent Behaviors are more accurately represented using a two-factor structure rather than a single-factor model in the Dutch context. Theoretically, this may suggest that Dutch young adults differentiate between distinct dimensions within these constructs in ways that diverge from the Canadian sample used in the original validation study (Humphreys & Brousseau, 2010).

The proposed two-factor model for Sexual Consent Norms suggests that Dutch young adults who show flexibility in consent norms as relationships become more committed do not necessarily extend that flexibility to norms around obtaining consent for specific sexual activities or their sequence. Based on the mean scores, participants appeared to hold stricter norms regardless of the activity type—indicating a belief that consent should be obtained for each individual sexual act, when compared to the relationship status. These distinctions align with findings from cross-cultural research showing that interpretations of consent-related behaviors vary significantly across cultural contexts (Beres, 2014). However, it is worth noting that the original English SCS-R was validated prior to the societal impact of the #MeToo movement, which may account for shifts in consent norms observed in the current study. To deepen our understanding of culturally rooted variations in perspectives on sexual consent we recommend conducting cross-cultural or cross-national comparative studies.

The suggested two-factor model for Indirect Consent Behaviors, which separates general nonverbal behaviors from partner-specific consent behaviors, is consistent with previous research indicating that the presence or absence of a partner, as well as relationship duration, are relevant factors influencing sexual consent communication (Muehlenhard et al., 2016). A methodological detail that may have contributed to this differentiation is the inclusion of a “Not Applicable” response option in the Dutch SCS-R for items referring to “my partner” or “my current (or most recent) partner”—a feature not present in the original English version. As a result, these items were only answered by respondents with current or past relationship experience. These findings underscore the need for further research into how sexual consent norms and behaviors differ between casual and ongoing relationships (Beres, 2014; Muehlenhard et al., 2016). We recommend retaining the “Not Applicable” response option in future Dutch SCS-R administrations when sampling individuals without current or past partners. Additionally, we suggest revising the phrasing of “My partner” to “My current (or most recent) partner” in Items 22, 33, and 34 to avoid forcing respondents to answer irrelevant questions and to reduce dropout attrition (Hochheimer et al., 2016). A version of the Dutch SCS-R reflecting these minor textual changes is provided in the Electronic Supplement (Table C).

Another noteworthy finding for this subscale was the relatively low reliability score of the scale assessing the use of indirect, nonverbal methods to communicate consent (Indirect Consent Behaviors), including its adapted form (Indirect Consent Behaviors: Nonverbal), in the Dutch version. This score was lower compared to both the other Dutch subscales and the reliability of the corresponding subscale in the original English version (Humphreys & Herold, 2007). A plausible explanation for this discrepancy may lie in cultural differences. In the Netherlands, verbally seeking sexual consent may be less normalized than in the Canadian context where the English version was validated. Notably, Canada introduced affirmative consent into its Criminal Code in 1982 in relation to sexual offenses (Shaffer, 2012). As a result, the perceived distinction between explicit and implicit expressions of sexual consent may be less pronounced among Dutch young adults. Future research is needed to develop culturally sensitive measures that accurately capture preferences for indirect and direct consent communication among Dutch young adults.

In line with this recommendation, it is important to emphasize that none of the CFA-based factor suggestions discussed above represent finalized improvements to the Dutch SCS-R. These adaptations require further refinement and empirical validation, particularly given the low internal consistency observed for two of the four proposed subscales. Nonetheless, the present findings provide a valuable foundation for future research into the flexibility of sexual consent norms and communication preferences in the Dutch context.

Lastly, the utilization of the SCS-R in samples comprising respondents with diverse gender identities and sexual orientations is supported by the results of the reliability scores in the total sample, along with comparable reliability scores and patterns in the gender and sexual minority subgroups (Griner et al., 2021). However, the discrepancies identified in the analyses exploring construct validity suggest that actual sexual consent experiences are significantly less aligned with sexual consent attitudes and intended behaviors among cisgender women and non-heterosexual individuals when compared to cisgender men and heterosexual individuals. These results are in line with previous findings that highlight more experiences with sexual assault among cisgender women and non-heterosexual individuals compared to cisgender men and heterosexuals, respectively (FRA, 2014; Martin‐Storey et al., 2018; Muehlenhard et al., 2017). To gain a deeper understanding of how this gap between attitudes, intentions, and experiences can be explained, future researchers are encouraged to include measures that distinguish between intentions and experiences concerning asking for and giving consent, as well as between verbal and nonverbal methods, as we could not make this distinction in our study (McKie et al., 2020; Walsh et al., 2019). Furthermore, research is needed to gain insight into effective interventions aimed at reducing sexual experiences without prior consent. For example, experimental studies could examine the effectiveness of inclusive sexual education programs designed to empower young people to respect the wishes and boundaries of others and to help them express their preferences related to sexual consent and activities (Epps et al., 2023; Rabbitte, 2020).

Several study limitations warrant acknowledgment. Firstly, not all gender identity and sexual orientation subgroups were sufficiently represented, resulting in relatively broad categories such as “non-heterosexual individuals.” Consequently, not all analyses could be performed within each subgroup, significantly limiting the ability to report differences among specific sexual and gender minority groups (Griner et al., 2021; McKie et al., 2020). Additionally, despite concerted recruitment efforts by the study team, respondents with a non-Western cultural background and those from religious groups other than Christianity were underrepresented. This underrepresentation limits the generalizability of the findings to these subgroups. Future studies should invest additional effort in identifying effective recruitment methods for these underrepresented populations in sexual consent research. This is particularly important, as culture and ideas about gender roles are expected to shape sexual consent attitudes and, ultimately, individuals’ behavior (Ahrold & Meston, 2010; Levand, 2020). Furthermore, due to the lack of other validated sexual consent questionnaires in Dutch, the exploratory construct validity analysis was based on the TPB. This analysis hypothesized that the SCS-R subscales predict intentions, which in turn predict behaviors (in this context, discussing sexual consent; Ajzen, 1991). However, considerable debate exists regarding the strength of the relationship between intentions and behavior (Sheeran & Webb, 2016), especially for behaviors involving more than one person, such as asking for and giving sexual consent during sexual encounters. Additionally, not all SCS-R subscales were included in the construct validity exploration. Therefore, the results of this initial exploration must be interpreted with caution, and future research is encouraged to supplement this analysis with other validation methods. Another limitation involves the risk of attrition bias, with approximately 19% of the 686 young adults who initiated the study not reaching the SCS-R questions. While this dropout rate aligns with expectations for relatively lengthy web-based surveys (Hoerger, 2010; Manfreda et al., 2008), it remains possible that specific subgroups, such as those with negative sexual experiences, discontinued participation more frequently. Given the extensive questionnaire—resulting from the collaboration of multiple research projects (approximately 140–160 items)—and the well-established association between survey length and respondent dropout (Hoerger, 2010), future studies should critically evaluate the survey’s length or consider counterbalancing item order to minimize its impact.

This study represents the first Dutch translation and initial validation of a measure designed to examine both attitudes and behaviors related to sexual consent within a diverse sample of young adults, encompassing variations in sexual orientation, gender identities, daily occupation, educational status, and religious backgrounds. The findings support the application of the Dutch SCS-R in future research aimed at exploring sexual consent-related attitudes, intentions, and awareness among young adults from diverse backgrounds. However, further research is needed to examine how the SCS-R can be used to explore the flexibility of norms, and indirect consent behaviors in the Dutch setting. In addition, cross-cultural and cross-country studies are encouraged to further examine possible culturally rooted differences. Notably, the observed gap between sexual consent-related preferences and actual experiences among cisgender women and non-heterosexual individuals highlights a critical area for future research. Understanding the factors contributing to this discrepancy is essential, as it may have important implications for sexual consent education, communication strategies, and interventions tailored to these groups. Addressing this gap could ultimately support the development of more inclusive and effective approaches to promoting healthy sexual consent practices.

## Supplementary Information

Below is the link to the electronic supplementary material.

Supplementary file1 (DOCX 222 KB)

## Data Availability

The data that support the findings of this study are not available due to local privacy policies.
