# Supplementary material for: Validation of the Dutch Sexual Consent Scale-Revised
Source: Arch Sex Behav. 2026 Apr 10;55(3):1309–23. doi: 10.1007/s10508-026-03415-3 (PMC13194265; doi:10.1007/s10508-026-03415-3)
Supplement: Supplementary file 1 — Supplementary file1 (DOCX 222 KB) [file 10508_2026_3415_MOESM1_ESM.docx]

**Validation of the Dutch Sexual Consent Scale-Revised**

**ELECTRONIC SUPLEMENT**

**Table A**

Demographic Information by Gender Identity

|  | Cisgender female^a^  (*n* = 378) | | Cisgender male^a^  (*n* = 150) | | Non-cisgender^a^  (*n* = 27) | |
| --- | --- | --- | --- | --- | --- | --- |
| Variable | *n* | % | *n* | % | *n* | % |
| Age group |  |  |  |  |  |  |
| 16 - 17 | 20 | 5 | 12 | 8 | 1 | 4 |
| 18 - 19 | 65 | 17 | 17 | 11 | 1 | 4 |
| 20 - 21 | 106 | 28 | 39 | 26 | 13 | 48 |
| 22 - 23 | 136 | 36 | 40 | 27 | 6 | 22 |
| 24 - 25 | 51 | 14 | 42 | 28 | 6 | 22 |
| Sex assigned at birth |  |  |  |  |  |  |
| Female | 378 | 100 | 0 | 0 | 18 | 67 |
| Male | 0 | 0 | 150 | 100 | 9 | 33 |
| Gender identity^a^ |  |  |  |  |  |  |
| Cisgender female | 378 | 100 | 0 | 0 | 0 | 0 |
| Cisgender male | 0 | 0 | 150 | 100 | 0 | 0 |
| Non-cisgender | 0 | 0 | 0 | 0 | 27 | 100 |
| [Sexual orientation](https://apastyle.apa.org/style-grammar-guidelines/bias-free-language/sexual-orientation) |  |  |  |  |  |  |
| Heterosexual | 250 | 66 | 117 | 78 | 6 | 22 |
| Heteroflexible | 24 | 6 | 3 | 2 | 0 | 0 |
| Bisexual | 44 | 12 | 11 | 7 | 5 | 19 |
| Lesbian/Gay | 10 | 3 | 13 | 9 | 5 | 19 |
| Questioning | 33 | 9 | 2 | 1 | 3 | 11 |
| Other^b^ | 17 | 4 | 4 | 3 | 8 | 30 |
| Relation status |  |  |  |  |  |  |
| Not in a relation | 178 | 47 | 87 | 58 | 15 | 56 |
| In a relation | 200 | 53 | 63 | 42 | 12 | 44 |
| Sexual experience^c^ |  |  |  |  |  |  |
| No | 64 | 17 | 39 | 26 | 7 | 26 |
| Yes | 314 | 83 | 111 | 74 | 20 | 74 |
| [Educational level](https://apastyle.apa.org/style-grammar-guidelines/bias-free-language/socioeconomic-status)^d^ |  |  |  |  |  |  |
| Level 1-4 | 86 | 23 | 48 | 32 | 4 | 15 |
| Level 5-8 | 292 | 77 | 102 | 68 | 23 | 85 |
| Housing  type |  |  |  |  |  |  |
| With parents | 154 | 41 | 76 | 51 | 8 | 30 |
| Student room/studio | 169 | 45 | 56 | 37 | 15 | 56 |
| Own (rental) house | 49 | 13 | 17 | 11 | 4 | 15 |
| Daily occupation |  |  |  |  |  |  |
| Education | 293 | 78 | 109 | 73 | 23 | 85 |
| Work | 81 | 21 | 39 | 26 | 4 | 15 |
| No work or education | 4 | 1 | 2 | 1 | 0 | 0 |
| [Cultural background](https://apastyle.apa.org/style-grammar-guidelines/bias-free-language/racial-ethnic-minorities)^e^ |  |  |  |  |  |  |
| Dutch/Western | 361 | 95 | 139 | 93 | 23 | 85 |
| Non-Western (+ Dutch) | 13 | 3 | 10 | 7 | 2 | 7 |
| Importance of religion |  |  |  |  |  |  |
| Not (at all) | 191 | 50 | 85 | 57 | 16 | 60 |
| A little | 94 | 25 | 23 | 15 | 8 | 30 |
| Very | 93 | 25 | 42 | 28 | 3 | 11 |

*Note.* The number of respondents and percentages do not sum up to 555/100% when the variable contained missing data.

^a^Cisgender females are respondents assigned female at birth and identify as a woman; ^a^Cisgender males are respondents assigned male at birth and identify as a man; Non-cisgender respondents identified as transgender females (*n* = 2), transgender males (*n* = 7), nonbinary (*n* = 4), Bi-gender (n=7), questioning (*n* = 6), and other (*n* = 1) respondents.

^b^The category “other” in Sexual Orientation included respondents that identified as pansexual (n=12), Queer (n=12), Asexual/demisexual (n=5).

^c^Sexual experience was operationalized as “actions that are intended to sexually stimulate someone, such as kissing, touching genitals, oral sex, or vaginal or anal sex”.

^d^International Standard Classification of Education (ISCED) educational level 1-4 = primary; lower secondary; upper secondary; post-secondary non-tertiary; and ISED educational level 5-8 = short cycle tertiary; bachelor or equivalent; master or equivalent; doctoral or equivalent.

^e^Cultural background categories are operationalized as: Dutch/Western = Only Dutch (*n =* 511), Dutch and North America (*n* = 1), Dutch and Western Europe (11); and “Non-Western (+ Dutch)” as Dutch and Turkish (*n =* 4), Dutch and Moroccan (*n =* 1), Dutch and Surinamese (*n =* 4), Dutch and Antillean (*n =* 2), Dutch and Europe non-Western (*n* = 3), Dutch and South/West Asia (*n* = 7), Dutch and North/East/Central Asia (*n* = 6), Dutch and not specified (*n* = 4)

| **Table B**  Sexual Consent Beliefs, Attitudes and Intentions (SCS-R) in Dutch |  |
| --- | --- |
| Items | Answer options^a^ |
| Subschaal 1: Positieve houding ten opzichte van seksuele toestemming |  |
| 1. Er moet altijd seksuele toestemming worden verkregen voordat je begint met seksuele activiteit. | 1-8 |
| 1. Het is altijd belangrijk om seksuele toestemming te vragen. Ook als je al een keer seks hebt gehad. | 1-8 |
| 1. Toestemming vragen voor seksuele activiteiten is in mijn eigen belang omdat het misverstanden kan voorkomen. | 1-8 |
| 1. Er moet eerst mondeling toestemming gevraagd worden, voordat je met seksuele activiteit begint. | 1-8 |
| 1. Wanneer je seksuele activiteit begint, moet je er altijd vanuit gaan dat je géén seksuele toestemming hebt. | 1-8 |
| 1. Het is net zo belangrijk om toestemming te vragen voor het aanraken van geslachtsdelen als voor vaginale en/of anale seks. | 1-8 |
| 1. De meeste mensen om wie ik geef, vinden dat ik altijd om seksuele toestemming zou moeten vragen. | 1-8 |
| 1. Je moet voor elke vorm van seksueel gedrag toestemming vragen. Dus ook voor kussen en aanraken. | 1-8 |
| 1. Als twee personen seks gaan hebben, zijn ze beide verantwoordelijk voor het vragen om toestemming voordat ze beginnen met seksuele activiteit. | 1-8 |
| 1. Je moet uitgaan van “nee” voordat je begint met seksuele activiteit, totdat je duidelijk toestemming voor seks hebt gekregen. | 1-8 |
| 1. Het is oké om soms geen seksuele toestemming te vragen. [R] | 1-8 |
| Subschaal 2: (Gebrek aan) ervaren gedragscontrole |  |
| 1. Ik zou het moeilijk vinden om seksuele toestemming te vragen, omdat het de stemming zou verpesten. | 1-8 |
| 1. Ik ben bang dat mijn partner me raar zou vinden als ik om seksuele toestemming zou vragen voordat we beginnen met seksuele activiteiten. | 1-8 |
| 1. Ik zou het moeilijk vinden om seksuele toestemming te vragen, omdat dit niet past bij hoe ik graag met seksuele activiteit begin. | 1-9 |
| 1. Ik zou me zorgen maken dat andere mensen mij vreemd vinden als ze wisten dat ik toestemming vraag voordat ik start met seksuele activiteit. | 1-9 |
| 1. Ik vind het ongemakkelijk als er mondeling om seksuele toestemming wordt gevraagd. | 1-8 |
| 1. Ik heb *niet* altijd om seksuele toestemming gevraagd (of toestemming gegeven), omdat ik het gevoel had dat het verkeerd zou uitpakken en we uiteindelijk geen seks zouden hebben. | 1-9 |
| 1. Het mondeling vragen om toestemming vermindert het plezier van de ontmoeting. | 1-8 |
| 1. Ik zou het moeilijk vinden om mijn seksuele toestemming uit te spreken, omdat ik daar te verlegen voor ben. | 1-8 |
| 1. Ik weet zeker dat ik om toestemming zou kunnen vragen aan een nieuwe seksuele partner. [R] | 1-8 |
| 1. Ik zou iemand niet om seksuele toestemming willen vragen, omdat het mij stil zou laten staan bij dat ik seksueel actief ben. | 1-8 |
| 1. Ik weet zeker dat ik om seksuele toestemming kan vragen aan mijn huidige seksuele partner. [R] | 1-9 |
| Subschaal 3: Normen over seksuele toestemming |  |
| 1. In een nieuwe relatie is het verkrijgen van seksuele toestemming nodiger dan in een vaste relatie. | 1-8 |
| 1. Bij een eenmalig seksueel contact is het verkrijgen van seksuele toestemming nodiger dan in een vaste relatie. | 1-8 |
| 1. Als een intieme relatie langer duurt is het vragen om seksuele toestemming minder nodig. | 1-8 |
| 1. Het is voldoende om alleen om toestemming te vragen als je begint met seksuele activiteiten. | 1-8 |
| 1. Alleen voor vaginale en anale seks is mondelinge toestemming nodig. | 1-8 |
| 1. Ik denk dat partners minder vaak seksuele toestemming vragen als een relatie langer duurt. | 1-8 |
| 1. Als iemand toestemming geeft voor vaginale of anale seks, dan kun je ervan uitgaan dat aanraken en strelen ook mag. | 1-8 |
| Subschaal 4: Indirecte benadering |  |
| 1. Meestal geef ik seksuele toestemming door mijn lichaamstaal (zonder woorden). | 1-9 |
| 1. Het is makkelijk om aan de lichaamstaal van de ander te zien of diegene wel of geen toestemming geeft voor seksuele activiteit. | 1-9 |
| 1. Ik vraag meestal om toestemming door iemand seksueel te benaderen en wacht dan op een reactie. Dan weet ik of ik door kan gaan of niet. | 1-9 |
| 1. Ik hoef *geen* seksuele toestemming aan mijn partner te vragen of geven, omdat mijn partner mij goed genoeg kent. | 1-9 |
| 1. Het geven of vragen van seksuele toestemming is *niet* nodig met mijn partner, omdat ik erop vertrouw dat mijn partner ´het goede doet´. | 1-9 |
| 1. Ik vraag altijd mondeling toestemming voordat ik begin met seksuele activiteit. [R] | 1-9 |
| Subscale 5: Awareness of sexual consent |  |
| 1. Ik heb wel eens met een vriend(in) gepraat over onderwerpen rondom seksuele toestemming. | 1-8 |
| 1. Ik heb leeftijdsgenoten horen praten over onderwerpen rondom seksuele toestemming. | 1-8 |
| 1. Ik heb wel eens met een partner over seksuele toestemming gepraat op een moment waarop we niet bezig waren met seks. | 1-9 |
| 1. Ik heb *niet* veel nagedacht over seksuele toestemming. [R] | 1-8 |

*Note.* Items with [R] are reverse coded.

^a^Answer options in Dutch: Helemaal oneens (1); Oneens (2); Een beetje oneens (3); Niet mee oneens en niet mee eens (4); Een beetje eens (5); Eens (6); Helemaal eens (7); Dat zeg ik liever niet (8); Niet van toepassing (9).

| **Table C**  Dutch SCS-R with the suggested Textual Alternations |  |
| --- | --- |
| Items | Answer options^a^ |
| Subschaal 1: Positieve houding ten opzichte van seksuele toestemming |  |
| 1. Er moet altijd seksuele toestemming worden verkregen voordat je begint met seksuele activiteit. | 1-8 |
| 1. Het is altijd belangrijk om seksuele toestemming te vragen. Ook als je al een keer seks hebt gehad. | 1-8 |
| 1. Toestemming vragen voor seksuele activiteiten is in mijn eigen belang omdat het misverstanden kan voorkomen. | 1-8 |
| 1. Er moet eerst mondeling toestemming gevraagd worden, voordat je met seksuele activiteit begint. | 1-8 |
| 1. Wanneer je seksuele activiteit begint, moet je er altijd vanuit gaan dat je géén seksuele toestemming hebt. | 1-8 |
| 1. Het is net zo belangrijk om toestemming te vragen voor het aanraken van geslachtsdelen als voor vaginale en/of anale seks. | 1-8 |
| 1. De meeste mensen om wie ik geef, vinden dat ik altijd om seksuele toestemming zou moeten vragen. | 1-8 |
| 1. Je moet voor elke vorm van seksueel gedrag toestemming vragen. Dus ook voor kussen en aanraken. | 1-8 |
| 1. Als twee personen seks gaan hebben, zijn ze beide verantwoordelijk voor het vragen om toestemming voordat ze beginnen met seksuele activiteit. | 1-8 |
| 1. Je moet uitgaan van “nee” voordat je begint met seksuele activiteit, totdat je duidelijk toestemming voor seks hebt gekregen. | 1-8 |
| 1. Het is oké om soms geen seksuele toestemming te vragen. [R] | 1-8 |
| Subschaal 2: (Gebrek aan) ervaren gedragscontrole |  |
| 1. Ik zou het moeilijk vinden om seksuele toestemming te vragen, omdat het de stemming zou verpesten. | 1-8 |
| 1. Ik ben bang dat mijn partner me raar zou vinden als ik om seksuele toestemming zou vragen voordat we beginnen met seksuele activiteiten. | 1-8 |
| 1. Ik zou het moeilijk vinden om seksuele toestemming te vragen, omdat dit niet past bij hoe ik graag met seksuele activiteit begin. | 1-9 |
| 1. Ik zou me zorgen maken dat andere mensen mij vreemd vinden als ze wisten dat ik toestemming vraag voordat ik start met seksuele activiteit. | 1-9 |
| 1. Ik vind het ongemakkelijk als er mondeling om seksuele toestemming wordt gevraagd. | 1-8 |
| 1. Ik heb *niet* altijd om seksuele toestemming gevraagd (of toestemming gegeven), omdat ik het gevoel had dat het verkeerd zou uitpakken en we uiteindelijk geen seks zouden hebben. | 1-9 |
| 1. Het mondeling vragen om toestemming vermindert het plezier van de ontmoeting. | 1-8 |
| 1. Ik zou het moeilijk vinden om mijn seksuele toestemming uit te spreken, omdat ik daar te verlegen voor ben. | 1-8 |
| 1. Ik weet zeker dat ik om toestemming zou kunnen vragen aan een nieuwe seksuele partner. [R] | 1-8 |
| 1. Ik zou iemand niet om seksuele toestemming willen vragen, omdat het mij stil zou laten staan bij dat ik seksueel actief ben. | 1-8 |
| 1. Ik weet zeker dat ik om seksuele toestemming kan vragen aan mijn huidige, of meest recente, seksuele partner. [R] | 1-9 |
| Subschaal 3: Normen over seksuele toestemming |  |
| 1. In een nieuwe relatie is het verkrijgen van seksuele toestemming nodiger dan in een vaste relatie. | 1-8 |
| 1. Bij een eenmalig seksueel contact is het verkrijgen van seksuele toestemming nodiger dan in een vaste relatie. | 1-8 |
| 1. Als een intieme relatie langer duurt is het vragen om seksuele toestemming minder nodig. | 1-8 |
| 1. Het is voldoende om alleen om toestemming te vragen als je begint met seksuele activiteiten. | 1-8 |
| 1. Alleen voor vaginale en anale seks is mondelinge toestemming nodig. | 1-8 |
| 1. Ik denk dat partners minder vaak seksuele toestemming vragen als een relatie langer duurt. | 1-8 |
| 1. Als iemand toestemming geeft voor vaginale of anale seks, dan kun je ervan uitgaan dat aanraken en strelen ook mag. | 1-8 |
| Subschaal 4: Indirecte benadering |  |
| 1. Meestal geef ik seksuele toestemming door middel van lichaamstaal (zonder woorden). | 1-9 |
| 1. Het is makkelijk om aan de lichaamstaal van de ander te zien of diegene wel of geen toestemming geeft voor seksuele activiteit. | 1-9 |
| 1. Ik vraag meestal om toestemming door iemand seksueel te benaderen en wacht dan op een reactie. Dan weet ik of ik door kan gaan of niet. | 1-9 |
| 1. Ik hoef *geen* seksuele toestemming aan mijn huidige, of meest recente, partner te vragen of geven, omdat deze partner mij goed genoeg kent. | 1-9 |
| 1. Het geven of vragen van seksuele toestemming is *niet* nodig met mijn huidige, of meest recente, partner, omdat ik erop vertrouw dat deze partner ´het goede doet´. | 1-9 |
| 1. Ik vraag altijd mondeling toestemming voordat ik begin met seksuele activiteit. [R] | 1-9 |
| Subscale 5: Awareness of sexual consent |  |
| 1. Ik heb wel eens met een vriend(in) gepraat over onderwerpen rondom seksuele toestemming. | 1-8 |
| 1. Ik heb leeftijdsgenoten horen praten over onderwerpen rondom seksuele toestemming. | 1-8 |
| 1. Ik heb wel eens met een partner over seksuele toestemming gepraat op een moment waarop we niet bezig waren met seks. | 1-9 |
| 1. Ik heb *niet* veel nagedacht over seksuele toestemming. [R] | 1-8 |

*Note.* Items with [R] are reverse coded.

^a^Answer options in Dutch: Helemaal oneens (1); Oneens (2); Een beetje oneens (3); Niet mee oneens en niet mee eens (4); Een beetje eens (5); Eens (6); Helemaal eens (7); Dat zeg ik liever niet (8); Niet van toepassing (9).
